# Screening of Mosquitoes for West Nile Virus and Usutu Virus in Croatia, 2015–2020

**DOI:** 10.3390/tropicalmed6020045

**Published:** 2021-04-02

**Authors:** Ana Klobucar, Vladimir Savic, Marcela Curman Posavec, Suncica Petrinic, Urska Kuhar, Ivan Toplak, Josip Madic, Tatjana Vilibic-Cavlek

**Affiliations:** 1 Department of Epidemiology, Andrija Stampar Institute of Public Health, 10000 Zagreb, Croatia; ana.klobucar@stampar.hr (A.K.); marcela.curman@stampar.hr (M.C.P.); suncica.petrinic@stampar.hr (S.P.); 2 Poultry Center, Croatian Veterinary Institute, 10000 Zagreb, Croatia; 3 Department of Virology, Institute of Microbiology and Parasitology, Veterinary Faculty, University of Ljubljana, 1000 Ljubljana, Slovenia; urska.kuhar@vf.uni-lj.si (U.K.); ivan.toplak@vf.uni-lj.si (I.T.); 4 Department of Microbiology and Infectious Diseases with Clinic, Faculty of Veterinary Medicine, University of Zagreb, 10000 Zagreb, Croatia; josip.madic@vef.hr; 5 Department of Virology, Croatian Institute of Public Health, 10000 Zagreb, Croatia; tatjana.vilibic-cavlek@hzjz.hr; 6 Department of Microbiology, School of Medicine, University of Zagreb, 10000 Zagreb, Croatia

**Keywords:** Usutu virus, West Nile virus, mosquitoes, Croatia

## Abstract

In the period from 2015 to 2020, an entomological survey for the presence of West Nile virus (WNV) and Usutu virus (USUV) in mosquitoes was performed in northwestern Croatia. A total of 20,363 mosquitoes were sampled in the City of Zagreb and Međimurje county, grouped in 899 pools and tested by real-time RT-PCR for WNV and USUV RNA. All pools were negative for WNV while one pool each from 2016 (*Aedes albopictus*), 2017 (*Culex pipiens* complex), 2018 (*Cx. pipiens* complex), and 2019 (*Cx. pipiens* complex), respectively, was positive for USUV. The 2018 and 2019 positive pools shared 99.31% nucleotide homology within the USUV NS5 gene and both clustered within USUV Europe 2 lineage. The next-generation sequencing of one mosquito pool (*Cx. pipiens* complex) collected in 2018 in Zagreb confirmed the presence of USUV and revealed several dsDNA and ssRNA viruses of insect, bacterial and mammalian origin.

## 1. Introduction

West Nile virus (WNV) and Usutu virus (USUV) are mosquito-borne arboviruses of the genus *Flavivirus*, family *Flaviviridae*. In Europe, both viruses have been detected in humans, horses, mosquitoes, and birds [1,2].

WNV is sustained in an enzootic cycle between birds and mosquitoes while humans and horses represent “dead-end” hosts. Numerous bird species are competent amplifier hosts for WNV, and mosquitoes of the genus *Culex* are the main vectors [3]. After the first isolation of WNV in Uganda (1937), sporadic WNV cases were recorded in the subsequent years. WNV has been responsible for sporadic outbreaks in Mediterranean countries since 1960s [4]. In the last decade, the occurrence of outbreaks in many European countries has significantly increased [1,5]. So far, the highest number of WNV infections in Europe was recorded in 2018. Number of cases were seven times higher than in 2017 (1605 versus 212) and exceeded all cases reported between 2011 and 2017 [6].

In Croatia, first human cases of WNV neuroinvasive disease (WNND) were reported in 2012 in eastern counties [7]. In the following years, small outbreaks as well as sporadic cases were continuously recorded in continental Croatian counties [5]. In addition to human cases, asymptomatic WNV infections and seropositivity in sentinel horses [8] as well as in poultry [9] were detected in the same geographic areas. In 2018, 54 cases of WNND and 7 cases of WNV fever were recorded in 11 Croatian continental counties. In the same year, WNV RNA was detected for the first time in two dead goshawks (*Accipiter gentilis*) from the same aviary in northwestern Croatia [10,11].

In Europe, the most important vector species of WNV are *Culex pipiens*, *Cx. perexiguus,* and *Cx. modestus* [12,13]. WNV has been isolated from several species in Europe: *Cx. pipiens*, *Ochlerotatus caspius,* and *Cx. modestus* in Italy [14,15,16,17]; *Cx pipiens* and *Cx. modestus* in Greece [18]; *Cx. pipiens* and *Cx. perexiguus* [19] in Spain; *Cx. modestus* and *Cx. pipiens* complex in Slovakia [20]; and *Cx. pipiens*, *Aedes vexans,* and *Culiseta annnulata* in Serbia [21].

USUV is a mosquito-borne arbovirus, genetically closely related to WNV. The natural cycle of USUV is similar to that of WNV and involves birds as the main amplifying hosts and *Culex* mosquitoes as the main vectors [22,23,24]. However, the virus has also been detected in mosquitoes from other genera within the family of Culicidae [22,25]. Humans and horses are incidental (dead-end) hosts of USUV as well. The first USUV isolation was in 1959 from *Cx. neavei* mosquito caught near the Usutu River in Swaziland [26]. Since then, the widespread circulation of the virus has been observed in many countries. In Europe, USUV emerged in 1996 in the Tuscany region (Italy) [27]. In the following years, until today, the virus has been detected in many European countries in birds, mosquitoes, horses, bats, and humans, indicating that USUV become endemic in Europe [22,25].

In Croatia, the first serologic evidence of USUV was reported in 2011 in two seropositive horses detected in northwestern Croatia [28]. In 2012, USUV neutralizing antibodies were found in a human sample from a resident of eastern Croatia. During the 2013 WNV outbreak, the first three cases of neuroinvasive USUV disease were detected in Zagreb and surrounding areas [29]. In 2018, during the largest WNV outbreak, three additional human cases of USUV neuroinvasive disease were detected in one northwestern and two eastern Croatian counties. Moreover, USUV RNA was detected for the first time in one dead blackbird (*Turdus merula*) in Zagreb County [5,25].

The first isolation of USUV in Europe was made in 2006 in *Cx. pipiens* mosquitoes from Catalonia, Spain [30]. Since then, the presence of USUV in mosquitoes was detected in several European countries in native and invasive mosquito species: *Cx. perexiguus* collected in southern Spain [19]; *Cx. pipiens*, *Ae. albopictus*, *Cs. annulata*, *Oc. detritus*, *Anopheles maculipennis* s.l., and *Oc. caspius* in Northern Italy [15,16,17,31,32,33]; *Cx. pipiens* in Serbia [34,35]; *Cx. pipiens/Cx. torrentium* and *Ae. japonicus* in Austria [36,37]; in overwintering *Cx. pipiens* pools in Belgium [38]; *Cx. modestus* in Czech Republic [39]; and *Cx. pipiens* in France [40] and Germany [41]. Although USUV has been found in several species, *Cx. pipiens* is the main vector for the virus [1,25].

Both viruses were most frequently detected in *Cx. pipiens* mosquitoes. Phylogenetic analysis of WNV strains from mosquitoes in different geographic areas in Europe showed circulation of WNV lineage 1 and 2 [2,5]. Analysis of USUV strains showed that the Europe 2 lineage is the most prevalent in mosquitoes; however, Europe 3 and 4 and Africa 2 and 3 lineages were also detected [25].

So far, 52 mosquito species have been detected in Croatia, of which two are invasive (*Ae. albopictus* and *Ae. japonicus*) [42]. In the Zagreb area, 32 mosquito species have been recorded so far [43,44], several species of them are potential vectors for arboviruses.

The first screening of mosquitoes for flaviviruses in Croatia was conducted during the 2012 WNV outbreak in three northeastern counties. Mosquitoes were sampled within the area where WNV human infections occurred and all tested *Cx. pipiens* complex pools were negative for WNV RNA [45]. The aim of this study was to detect the presence of WNV and USUV in mosquitoes in northwestern Croatian regions. Virus screening of mosquitoes started in 2015 and has been continuously carried out until the end of 2020.

## 2. Materials and Methods

### 2.1. Mosquito Collection and Identification

The research area was the northwestern part of Croatia. It included the City of Zagreb and Međimurje County. WNV and USUV were previously identified in that Croatian region, based on testing of human samples, horses, poultry and wild birds [10,29].

In the period from 2015 to 2020 a total of 618 mosquito sampling occasions were conducted using three methods (Table 1), 613 in the City of Zagreb, and 5 in Međimurje County.

**Figure 1:**
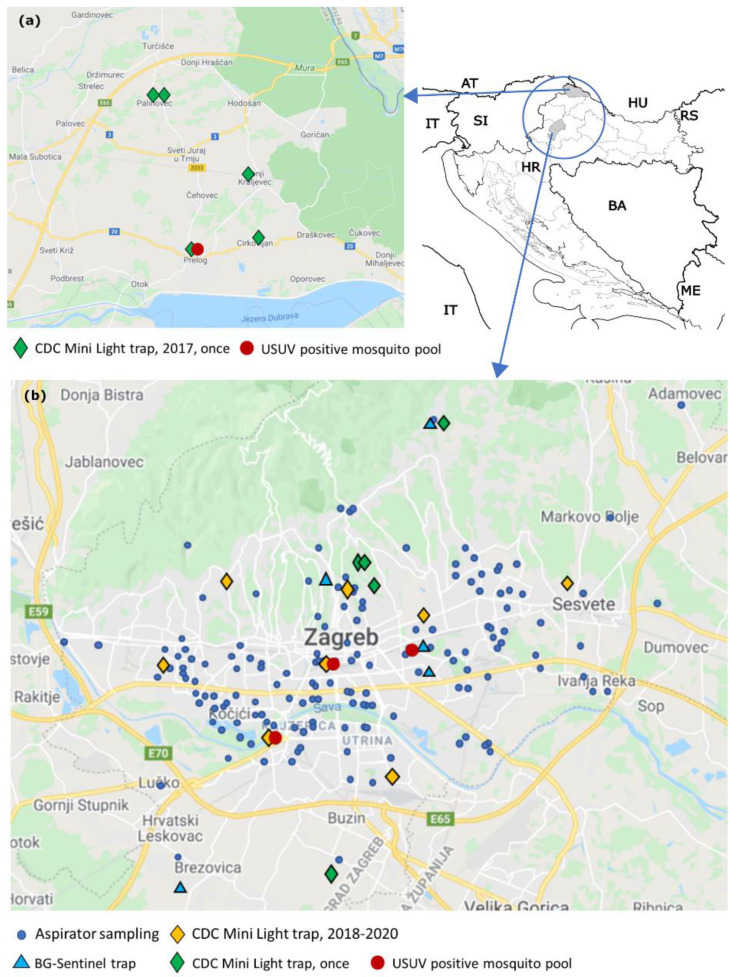
Distribution of mosquito sampling locations and Usutu virus (USUV) positive mosquito pools in northwestern Croatia, (**a**) Međimurje County, 2017; (**b**) City of Zagreb, 2015–2020.

In the area of the City of Zagreb (641,355 square kilometers; 804,507 inhabitants), the mosquito sampling occasions were conducted over six years (2015−2020). Mosquitoes were collected by three different traps and methods, including CDC Mini Light traps (BioQuip, Products, Rancho Dominguez, CA, USA), BG-Sentinel traps (Biogents, Germany), and aspirator collection. CDC Mini Light traps were equipped with dry ice (CO_2_) as an attractant (CO_2_-baited traps) and used to collect adult mosquitoes of various species. They were placed approximately 1.5 m from the ground and set in the late afternoon, before sunset, left overnight, and removed after sunrise (07:00–10:00). Over the last three years (2018–2020), the CO_2_-baited traps were set at the same eight collection sites (Figure 1b, yellow marks), every 14 days, from May to October. A total of 264 sampling occasions were gathered (2018: 72; 2019: 96; 2020: 96) (Table 1). The following habitats were chosen for sampling: woods (three locations), a populated area close to the green belt (two locations), gardens in the urban part of the city (two locations), city center close to Zagreb botanical garden (one location). Additionally, during the research, the sampling occasions using CO_2_-baited trap were conducted once at five collection sites (Figure 1b, green marks). During all years, mosquito samples have been collected by aspirator as well as using the human landing collection method: during field surveys in the yards of citizens who complained of mosquitoes, on open area in public places (parks, cemeteries, green areas), from the walls in underground shelters, flats, and cellars (Figure 1b, blue dots). In the period between 2015 and 2017, most mosquito samples were collected using an aspirator. During six years of research, a total of 331 sampling occasions by an aspirator were gathered. In 2015, BG-Sentinel traps with BG Lure attractant were used for collecting *Ae. albopictus*. The sampling was performed during the afternoon at five sites (Figure 1b, light blue marks) two or three times, with a total of 13 occasions.

In Međimurje County (the northernmost Croatian county; 729.58 square kilometers; 113,804 inhabitants), mosquito samples were collected only once, between August 17 and 18 2017, from the late afternoon to the morning, at five different locations (Figure 1a, green marks) using traps with CO_2_. Four traps were placed next to chicken farms and next to a horse stable.

The mosquitoes sampled by traps with CO_2_ were transported to the laboratory in containers with dry ice, transferred to plastic tubes, and stored on dry ice until identification. Mosquitoes trapped by the aspirator were transported alive to the laboratory in aspirator, placed briefly in a freezer at −18 °C, then identified. Female mosquitoes were morphologically identified by species or species complex on a chilling surface under a stereomicroscope, using the determination keys by Becker et al. (2010) [46] and Schaffner et al. (2001) [47]. Specimens belonging to the same species/complex collected on the same day and at the same sampling site were pooled, with up to 60 individuals per pool, and stored at −80 °C until virological testing.

### 2.2. Virological Testing

Viral RNA was extracted using a High Pure Viral Nucleic Acid Kit (Roche Applied Science). TaqMan real-time RT-PCR assays for detection of WNV and USUV RNA were performed according to the protocols of Tang et al. (2006) [48] and Nikolay et al. (2014) [49], respectively, using a Brilliant III Ultra-Fast QPCR Master Mix (Agilent Technologies) and a Rotor-Gene Q real-time PCR cycler (Hilden, Germany). Samples identified as positive using the real-time RT-PCR assays were tested by conventional RT-PCR using PrimeScript™ One Step RT-PCR Kit Ver.2 (Takara Bio Inc, Kusatsu, Japan) and panflavi primers targeting the NS5 gene (FP: 5′-TACAACATGATGGGVAARAGAGAGA-3′, RP: 5′-AGCATGTCTTCYGTBGTCATCCAYT-3′) to amplify 1085 bp (WNV) and 1084 bp (USUV) products according to the protocol of Weissenböck et al. (2002) [50]. After electrophoresis, DNA samples extracted from excised gel fragments were Sanger sequenced in both directions by Humanizing Genomics, Macrogen Inc. with the use of the same primers. Genotyping and phylogenetic grouping of obtained sequences were based on comparison with strains retrieved from GenBank and obtained using BlastN algorithm (http://www.ncbi.nlm.nih.gov (accessed on 28 February 2021)). Maximum likelihood phylogenetic analysis was conducted, and an evolutionary analysis was performed by using MEGA7 [51].

Ion Torrent high-throughput next-generation sequencing (NGS) technology was used to determine the virome in one selected *Cx. pipiens* pool. The RNA was extracted from sample suspensions with Trizol reagent (Invitrogen, Carlsbad, CA, USA). The cDNA was synthesized from the extracted nucleic acids using the cDNA Synthesis System (Roche, Manheim, Germany) and Random Hexamer Primers (Roche, Manheim, Germany) and fragmented with a Covaris M220 targeting peak fragment lengths of 400 bp. The library was prepared with the GeneRead™ DNA Library L Prep Kit (Qiagen, Hilden, Germany), according to the manufacturer’s instructions and sequenced on the Ion PGM platform using the Ion PGM™ Hi-Q™ View Sequencing Kit reagents (ThermoFisher Scientific–Ion Torrent, CA, USA). Sequenced reads were quality checked and trimmed using the Ion Torrent Suite v.5.6.0. Additionally, low quality bases were trimmed with Geneious software suite v.11.0.5 (Biomatters Ltd., Auckland, New Zealand). Clean reads were subjected to BlastX and BlastN search. The BlastX and BlastN results were analyzed with MEGAN6 [52] for the taxonomic assignment of the reads using the Lowest Common Ancestor (LCA) algorithm. To confirm the BlastX and BlastN results and to determine the exact number of reads belonging to the Blast identified RNA viruses, all clean reads were mapped against these RNA virus genomes with the Geneious reference mapper (Geneious software suite v.11.0.5, Biomatters Ltd., Auckland, New Zealand).

## 3. Results

During a six-year period (2015–2020) a total of 20,363 mosquitoes were collected and identified in northwestern Croatia, of which 20,291 from the City of Zagreb, and 72 mosquitoes from Međimurje County. The mosquitoes belong to 11 species. Of these, in the City of Zagreb, 31.1% of mosquitoes were identified as *Oc. sticticus*, followed by *Ae. albopictus* (23.5%), *Ae. vexans* (21,5%), and *Cx. pipiens* (20.7%). Other species were represented by less than 2%. Out of a total of 72 mosquitoes from Međimurje County, 52.8% belong to *Cx. pipiens* complex, followed by *Ae. vexans* (41.7%) and *Oc. sticticus* (5.5%) (Table 2).

Mosquito specimens were sorted in 899 pools and tested for the presence of WNV and USUV, 893 from the City of Zagreb and 6 from Međimurje County (Table 3). All tested mosquito pools were negative for WNV. A total of three USUV-positive pools were detected in Zagreb (one Ae. albopictus pool in 2016, one Cx. pipiens complex pool in 2018 and one 2019, respectively) and one *Cx. pipiens* complex pool in 2017 from Međimurje County (Prelog). All USUV positive mosquitoes were trapped between July 19 and September 7. The USUV-positive *Ae. albopictus* pool contained 15 mosquitoes which were collected by human landing collection on September 2 and September 7, 2016, in two adjacent yards in Zagreb. The other three positive pools comprised *Cx. pipiens* complex mosquitoes collected by the CO_2_-baited trap. In 2017, one pool of mosquitoes collected on August 19 in Prelog (Međimurje County) next to a horse stable. In 2018, a positive pool was trapped on July 19 in Zagreb, in a wood near the Jarun lake. The last *Cx. pipiens* complex positive pool was collected on 1 August 2019, in the center of Zagreb, near the Botanical garden (Figure 1, red dots).

**Figure 2:**
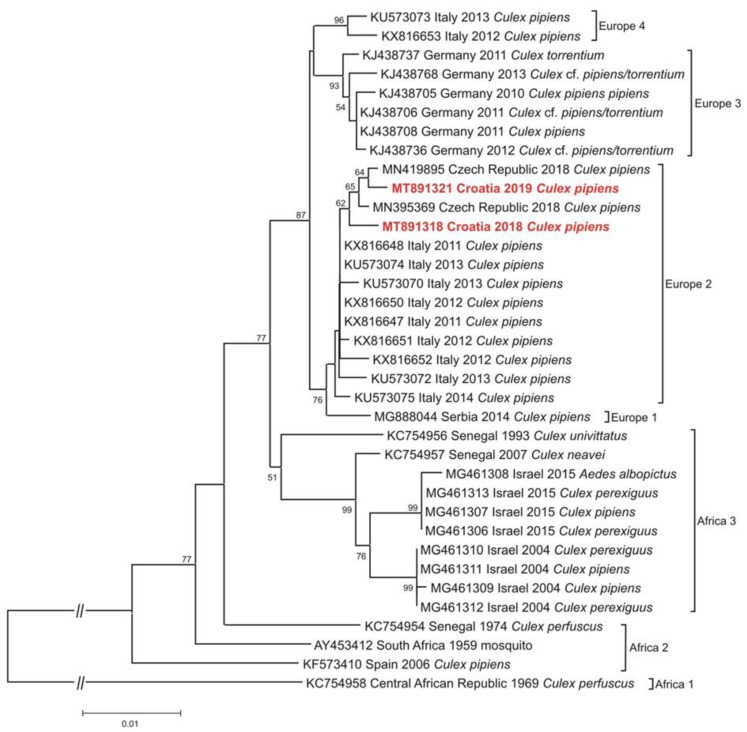
Phylogenetic neighbor-joining analysis of a 1015 nucleotide fragment of the USUV NS5 gene (corresponding nucleotide positions 9072–10,088 of the SAAR-1776 strain, GenBank accession numberAY453412) detected in *Culex pipiens* pool in Croatia and representative USUV strains from mosquitoes (n = 36). GenBank accession numbers, countries of origins, isolation/detection years and mosquito species are indicated at the branches. Viruses from Croatia are marked in bold and red color. USUV genetic lineages suggested by Cadar et al. (2017) [38] are indicated on the right. Supporting (≥50%) bootstrap values of 1000 replicates are displayed at the nodes. Horizontal distances are proportional to genetic distance. Scale bar indicates nucleotide substitutions per site. The interrupted branches, indicated by double slashes, were shortened by 80% for better graphic representation.

Conventional RT-PCR yielded a USUV positive result for *Cx. pipiens* pools collected in 2018 and 2019, and both PCR products were successfully sequenced. Both detected nucleotide sequences of the NS5 gene clustered within the USUV Europe 2 lineage (Figure 2) with 99.31% pairwise identity between each other. The 2018 USUV showed the highest similarity (99.71% nucleotide homology) with USUV detected in blackbirds (*Turdus merula*) in Austria in 2016 (accession number MF063042) and Czech Republic in 2017 (accession number MN419913), respectively. In contrast, the 2019 USUV detection shared the highest nucleotide homology (99.71%) with USUV detected in 2016 in *Cx. modestus* mosquito from Czech Republic.

The previously determined RT-PCR USUV positive sample, which consisted of *Cx. pipiens* (collected near the Jarun lake Zagreb, 2018), was whole virome sequenced by using the NGS method and a total of 817,573 (mean length of 184 nucleotides) clean reads were obtained from the sample. The BlastX and BlastN search identified 16,888 reads (2.7% of total reads) belonging to viruses. Other reads belonged to cellular organisms, to other sequences and to unassigned sequences. Most of the virus sequences were assigned to viruses infecting mosquitoes. Virus sequences belonging to six different virus families and to unclassified viruses were identified, including dsDNA and ssRNA viruses of insect, bacterial and mammalian origin (Table 4). Bacteriophages belonging to family Myoviridae (39 reads) and insect viruses belonging to Nudiviridae (64 reads), which are dsDNA viruses, were detected in the sample. The ssRNA (+) virus sequences were predominantly from *Culex* picorna-like virus 1 (12,067 reads), sequenced in complete genome within Picornaviridae family. A closely related strain in Genbank was *Culex* picorna-like virus 1 strain 16-0168/ROK/2016 (MH703059) with 96.56% nucleotide identity to the determined virus. Other identified ssRNA (+) virus sequences revealed the complete genome (2409 reads) of Alphamesonivirus 1 with 99.36% nucleotide identity with Alphamesonivirus 1 strain 11/2008 (MF281710) from the Mesoniviridae family. The identified *Culex* mosquito virus 4 (1171 reads) which belongs to the family Nodaviridae and had 96.03% nucleotide identity with strain CMos/Santa Clara (MH188031). The presence of USUV was also confirmed (31 reads). Virus sequences with most homology to an unclassified ssRNA (−) virus (916 reads) and to an unclassified RNA virus (191 reads), both of mosquito origin, were also detected in the sample.

## 4. Discussion

Human neuroinvasive USUV infections in Croatia were reported in 2013, and the first detection of USUV in mosquitoes was recorded in 2016. The USUV positive *Ae. albopitus* pool comprising 15 mosquitoes was collected in Zagreb, the same area where the first human cases were detected in 2013, i.e., two years before the start of the mosquito screening in Croatia. In 2017, there was a small WNV outbreak involving 8 human cases from five Croatian counties. Human USUV infections were not reported at that time; nevertheless, one pool of *Cx. pipiens* complex mosquitoes collected in north-west Croatia (Prelog, Međimurje County) tested positive for USUV RNA. In addition, one pool of *Cx. pipiens* complex mosquitoes collected in Zagreb in 2018 also tested positive for USUV RNA. In 2018, additional three cases of human neuroinvasive USUV infections were reported during the largest WNV outbreak in Croatia. One USUV infected patient was a resident of Zagreb County. Furthermore, USUV RNA was detected for the first time in one dead blackbird (*Turdus merula*) from the same geographic area (Zagreb County) [10]. USUV infections were not reported in humans and animals in Croatia since 2018. However, in 2019, USUV RNA was detected in one *Cx. pipiens* complex pool collected in Zagreb. Phylogenetic analysis of NS5 gene of USUV detected in the two *Cx. pipiens* pools collected in 2018 and 2019, respectively, showed their high nucleotide homology (99.31%), both of them clustering within USUV Europe 2 lineage. Detected sequences from a fatal human case (north-east Croatia, 2018) and a dead blackbird (north-west Croatia, 2018) also belonged to the USUV Europe 2 lineage [10]. This indicates USUV Europe 2 lineage endemization in Croatia. Further, each of the USUV sequences from the mosquito pools have shown high nucleotide homology (99.71%) with USUV strains from Czech Republic and Austria detected in 2016 and 2017. Unfortunately, the USUV positive mosquito pools detected in Croatia in 2016 and 2017 could not be successfully sequenced most probably due to the low amount of the virus in the samples (Ct value > 30, data not shown). Nevertheless, the USUV Europe 2 lineage endemization in Croatia should be observed in the context of the high prevalence of this lineage in other Central European countries [36,39].

Although outbreaks or sporadic WNV infections as well as seropositivity in humans and sentinel animals (horses and poultry) have been continuously recorded in continental Croatian counties [5,10,11], WNV RNA was not detected in any of the tested mosquito pools. This could be explained by targeted virological testing of symptomatic patients, sick or dead animals and particularly by serological testing of humans and sentinel animal in contrast to random virological testing of mosquitoes. Even though a large number of mosquitoes was tested, it was obviously underrepresented in terms of the WNV activity monitoring. This means that screening of mosquitoes for WNV has shown to be less sensitive than monitoring of WNV activity using sentinel animals.

In the Zagreb area, 32 species of mosquitoes have been recorded so far, two of them are invasive species, *Ae. albopictus* and *Ae. japonicus* [43,44]. *Aedes albopictus* is established in Zagreb and neighboring counties [53]. The presence of USUV in mosquitoes in Zagreb was first detected in the *Ae. albopictus* pool, whereas the other USUV positive pools were *Cx. pipiens* mosquitoes. Of all the tested pools, 25% were *Ae. albopictus* and 21% *Cx. pipiens* complex. *Aedes albopictus* is an invasive species that is rapidly adapting to temperate regions, already known as a vector of dengue, chikungunya, Zika and other arboviruses [54,55]. It is highly anthropophilic but also has a tendency to feed on different hosts, including birds, which significantly increases the potential role of the species in USUV transmission cycle [55]. Although *Ae. albopictus* seems to have low vector competence for USUV [56], numerous *Ae. albopictus* pools sampled through the surveillance program of the Emilia-Romagna region (Northern Italy) in the period 2009–2012 tested positive to this virus [16,31,32]. Taken together with the USUV positive *Ae. albopicus* pool in this study, it is necessary to monitor the development of this vector-pathogen association.

The *Cx. pipiens* complex includes two morphologically indistinguishable biotypes, *Cx. pipiens* biotype *pipiens* and *Cx. pipiens* biotype *molestus*. Members of the *Cx. pipiens* complex, as well as their hybrids, represent the most important vectors of arboviruses including WNV and USUV. In the city of Zagreb, the presence of both biotypes and their hybrids is confirmed by DNA barcoding (unpublished data, project “DNA Barcoding of Diversity of Croatian Fauna”). During the research in Zagreb and Međimurje area, *Cx. pipiens* complex individuals were sampled by CO_2_-baited trap and by aspirator from walls in apartments, underground shelters, and cellars. Approximately 20 pools were overwintering mosquitoes collected during November, December, and January in underground shelters and cellars. All three USUV positive *Cx. pipiens* pools were sampled in open area by CO_2_-baited trap and all overwintering mosquitoes were negative. Nevertheless, the high nucleotide homology (99.31%) between two USUV sequences found in *Cx. pipiens* in 2018 and 2019 in Zagreb raises question on USUV overwintering in mosquitoes in Croatia.

The NGS results of a *Cx. pipiens* pool revealed, beside USUV, several dsDNA (Myoviridae, Nudiviridae) and ssRNA (Picornaviridae, Mesoniviridae, Nodaviridae) viruses as well as two unclassified RNA viruses, namely, Hubei chryso-like virus 1 and Wuhan Mosquito Virus 8. Although these viruses were not our primary research aim, this finding presents a contribution to the knowledge on a geographical variation of the viromes in medically important mosquito vectors.

## 5. Conclusions

Detection of USUV in mosquitoes during the four consecutive transmission seasons (2016–2019) indicates the virus has become endemic in northwestern Croatia. Although WNV infections in humans and sentinel animals have been continuously recorded, WNV has not been detected in mosquitoes in Croatia so far.

## Figures and Tables

**Table 1:** Number of mosquito collecting using different methods at all sites.

Method of Collecting	2015	2016	2017	2018	2019	2020	Total
CO_2_-baited trap			7	75	96	96	274
Aspirator	116	23	57	41	43	51	331
BG Sentinel	13						13
Total	129	23	64	116	139	147	618

**Table 2:** Number of collected mosquitoes by species and sampling method.

Mosquito Species	Sampling Method	Number of Collected Specimens per Species								
		2015	2016	2017	2018	2019	2020	Subtotal	Total	%
City of Zagreb										
*Aedes albopictus*	Aspirator	1231	455	534	96	115	572	3003	4768	23.49
	CO_2_-baited trap				380	619	537	1536		
	BG Sentinel	229						229		
*Aedes cinereus*	Aspirator	2		1	13	2		18	67	0.33
	CO_2_-baited trap					49		49		
*Aedes rossicus*	Aspirator					18		18	338	1.67
	CO_2_-baited trap					320		320		
*Aedes vexans*	Aspirator	38		17		144	266	465	4359	21.48
	CO_2_-baited trap			1	189	2290	1414	3894		
*Aedes japonicus*	Aspirator			2				2	2	0.01
*Coquillettidia richiardii*	CO_2_-baited trap						8	8	8	0.04
*Culex modestus*	CO_2_-baited trap					1		1	1	0.005
*Culex pipiens* complex	Aspirator	475		53	345	2	257	1132	4210	20.75
	CO_2_-baited trap			20	1144	946	906	3016		
	BG Sentinel	62						62		
*Ochlerotatus geniculatus*	CO_2_-baited trap				78	81		159	159	0.78
*Ochlerotatus rusticus*	Aspirator				71			71	71	0.35
*Ochlerotatus sticticus*	Aspirator	327		115	848	506	82	1878	6308	31.09
	CO_2_-baited trap				584	3541	305	4430		
Total		2364	455	743	3670	8631	4428	20,291	20,291	100.00
**Međimurje County**										
*Culex pipiens* complex	CO_2_-baited trap			38				38		52.77
*Aedes vexans*	CO_2_-baited trap			30				30		41.66
*Ochlerotatus sticticus*	CO_2_-baited trap			4				4		5.55
Total				72				72	72	100.00

**Table 3:** Number of mosquito pools tested for the presence of West Nile virus and Usutu virus RNA by real-time RT-PCR.

Mosquito Species	2015	2016	2017	2018	2019	2020	Total
City of Zagreb							
*Aedes albopictus*	42	19 *	27	32	49	54	223
*Aedes cinereus*	1			2	9		12
*Aedes rossicus*					17		17
*Aedes vexans*	4		9	15	99	62	189
*Aedes japonicus*			1				1
*Coquillettidia richiardii*						2	2
*Culex modestus*					1		1
*Culex pipiens* complex	18		7	62 *	55 *	45	187
*Ochlerotatus geniculatus*					6	7	13
*Ochlerotatus rusticus*				9			9
*Ochlerotatus sticticus*	14		9	57	136	23	239
Total	79	19	52	177	372	193	893
**Međimurje County**							
*Culex pipiens* complex			3 *				3
*Aedes vexans*			2				2
*Ochlerotatus sticticus*			1				1
Total			6				6

* Single pool tested positive for the presence of USUV RNA.

**Table 4:** Viruses detected by NGS in a *Cx. pipiens* complex mosquito pool sampled in Zagreb, 2018.

Group	Order	Family/Genus (Species)	Number of Reads	Host
dsDNA	Caudovirales	Myoviridae	39	Bacteria
	/	Nudiviridae	64	Insects
ssRNA	Picornavirales	Picornaviridae (*Culex* picorna-like virus 1)	12,067	Mosquitoes
	Nidovirales /	Mesoniviridae/*Alphamesonivirus* (Alphamesonivirus 1) Nodaviridae (*Culex* mosquito virus 4)	2409 1171	Mosquitoes Mosquitoes
	/	Flaviviridae/*Flavivirus* (Usutu virus)	31	Mammals and birds
	/	Unclassified RNA virus (Hubei chryso-like virus 1)	191	Mosquitoes
	/	Unclassified RNA virus (Wuhan Mosquito Virus 8)	916	Mosquitoes
